# Impact of the Spectral Composition of Kilovoltage X-rays on High-Z Nanoparticle-Assisted Dose Enhancement

**DOI:** 10.3390/ijms22116030

**Published:** 2021-06-02

**Authors:** Maria A. Kolyvanova, Alexandr V. Belousov, Grigorii A. Krusanov, Alexandra K. Isagulieva, Kirill V. Morozov, Maria E. Kartseva, Magomet H. Salpagarov, Pavel V. Krivoshapkin, Olga V. Dement’eva, Victor M. Rudoy, Vladimir N. Morozov

**Affiliations:** 1 State Research Center-Burnasyan Federal Medical Biophysical Center of Federal Medical Biological Agency, 123098 Moscow, Russia; kolyvanova@physics.msu.ru (M.A.K.); belousovav@physics.msu.ru (A.V.B.); krusanov@physics.msu.ru (G.A.K.); kia2303@yandex.ru (A.K.I.); magometonco@mail.ru (M.H.S.); 2 Emanuel Institute of Biochemical Physics, Russian Academy of Sciences, 119334 Moscow, Russia; 3 Gause Institute of New Antibiotics, 119021 Moscow, Russia; 4 Department of Physics, Lomonosov Moscow State University, 119234 Moscow, Russia; morozov.kv15@physics.msu.ru; 5 Frumkin Institute of Physical Chemistry and Electrochemistry, Russian Academy of Sciences, 119071 Moscow, Russia; maryakar@mail.ru (M.E.K.); redmoun@mail.ru (O.V.D.); dema_ol@mail.ru (V.M.R.); 6 SCAMT Institute, ITMO University, 191002 St. Petersburg, Russia; krivoshapkin@scamt-itmo.ru

**Keywords:** radiotherapy, nanoparticles, X-ray, dose enhancement factor, radiosensitization, Monte-Carlo simulation

## Abstract

Nanoparticles (NPs) with a high atomic number (*Z*) are promising radiosensitizers for cancer therapy. However, the dependence of their efficacy on irradiation conditions is still unclear. In the present work, 11 different metal and metal oxide NPs (from Cu (*Z*_Cu_ = 29) to Bi_2_O_3_ (*Z*_Bi_ = 83)) were studied in terms of their ability to enhance the absorbed dose in combination with 237 X-ray spectra generated at a 30–300 kVp voltage using various filtration systems and anode materials. Among the studied high-*Z* NP materials, gold was the absolute leader by a dose enhancement factor (DEF; up to 2.51), while HfO_2_ and Ta_2_O_5_ were the most versatile because of the largest high-DEF region in coordinates U (voltage) and E_eff_ (effective energy). Several impacts of the X-ray spectral composition have been noted, as follows: (1) there are radiation sources that correspond to extremely low DEFs for all of the studied NPs, (2) NPs with a lower *Z* in some cases can equal or overcome by the DEF value the high-*Z* NPs, and (3) the change in the X-ray spectrum caused by a beam passing through the matter can significantly affect the DEF. All of these findings indicate the important role of carefully planning radiation exposure in the presence of high-*Z* NPs.

## 1. Introduction

The broad possibilities of using nanomaterials in nuclear medicine and radiation therapy have been shown in recent decade. They can be utilized in a wide variety of applications, from ionizing radiation generation [1] and dosimetry [2] to the targeted delivery of radionuclides [3] and tumor imaging [4]. The use of nanomaterials for modifying the biological effect of ionizing radiation is also a hopeful approach to increase the efficacy of cancer treatment. For example, nanoparticles (NPs) with a high atomic number (*Z*) are promising antitumor radiosensitizers capable of the local enhancement of the absorbed dose because of the large cross section of interactions with electromagnetic ionizing radiation [5,6,7,8]. In particular, NPs can be used when combining irradiation with other therapeutic modalities [9]. NPs whose *Z* (or effective atomic number *Z_eff_* in the case of multielement NPs) significantly exceed ones for the soft tissues (e.g., NPs based on bismuth (*Z*_Bi_ = 83), gold (*Z*_Au_ = 79), tantalum (*Z*_Ta_ = 73), silver (*Z*_Ag_ = 47), etc.) are attractive. Since the pioneering work by Hainfeld et al. [10] 16 years ago, plenty of NPs of various compositions, sizes, and shapes have been studied under different conditions (in silico, in vitro, and in vivo) [11,12,13,14,15]. Currently, NP preparations based on hafnium (*Z*_Hf_ = 72) and gadolinium (*Z*_Gd_ = 64) are undergoing clinical trials [16,17].

High-Z NPs radiosensitization is based on the absorption of ionizing radiation energy. Therefore, the irradiation conditions can strongly affect NPs efficacy. From the entire range of clinically relevant photon energies (from 30 keV to 25 MeV), the highest NPs radiosensitization effect corresponds to the region of up to ≈300 keV, because of the maximum photoelectric absorption ($\sigma_{PE}\sim Z^{3÷5}$). These energies are usually used for the treatment of superficial neoplasms and, less often, for internal irradiation [18,19,20]. For example, Kong et al. [21] observed an enhanced death of MCF-7 cells upon 220 kVp irradiation in the presence of gold NPs (GNPs) modified with cysteamine or thioglucose, while no significant effect was found with ^60^Co (≈1.25 MV) and ^137^Cs (≈0.662 MV) radiation. The radiosensitization efficacy of GNPs under the irradiation of MDA-MB-231 cells with 160 kVp photons, as found by Jain et al. [22], exceeded the efficacy obtained using 6 MeV and 15 MeV photons. A remarkable example of the dependence of the NPs radiomodifying effect on photon energy is a work by Briggs et al. [23], who showed the ability of cerium oxide NPs to exhibit radioprotection under irradiation with 10 MeV bremsstrahlung photons and to enhance radiation-induced cell death with 150 kVp X-ray treatment.

High-*Z* NPs can demonstrate different radiosensitization efficacies in the kilovoltage energy range. The sensitizer enhancement ratio (SER) values may vary widely (for convenience, we separated SER (sensitizer enhancement ratio; the indicator of “biological” effect) and DEF (dose enhancement factor; the indicator of macroscopic “physical” effect)). For example, SERs of about 1.1–1.8 are the most often observed for GNPs [24]. At the same time, significantly larger values (up to ≈24.6) are known [25]. In addition, despite the ability of CeO_2_ NPs to absorbed dose enhancement in the keV energy range [26], in a number of works, they demonstrated a radioprotective effect with 200 kVp treatment [27,28]. Such differences can be related, among other physico-chemical and biological factors (e.g., various design of used NPs, different cell lines, and different studied dose rates), to the features of the spectral composition of the X-ray radiation of various machines. As X-ray tubes, the traditional sources used in both therapeutic and research equipment, may have hugely different characteristics (voltage range, anode material, filtration system, etc.), the radiation they generate can differ significantly in the energy spectrum. Comparisons of the absorbed dose enhancement and/or the radiosensitization efficacy for high-*Z* NPs have been performed in several previous studies for a quite small number of X-ray spectra [29,30,31,32,33,34]. However, the systematic study of the influence of the X-ray spectral composition, as well as the effect of beam attenuation caused by the tissue penetration of X-rays, has not yet been carried out.

Such an investigation was the main goal of the present work. To reach it, a set of more than 230 different kilovoltage X-ray spectra was used. Another aim of the work was to compare the efficacy of NPs with different compositions. Thus, the NPs of a number of high-*Z* metals (Cu, Ag, Pt, and Au) and metal oxides (ZrO_2_, CeO_2_, Gd_2_O_3_, Tm_2_O_3_, HfO_2_, Ta_2_O_5_, and Bi_2_O_3_) were chosen, most of which have already been studied in combination with radiation [23,34,35,36,37,38,39,40]. The efficacy of the promising compositions of high-*Z* NPs was evaluated in terms of a macroscopic enhancement of the absorbed dose. In this approach, the presence of NPs was set by changing the mass composition of virtually irradiated matter, similarly to [26,29,34,40,41].

## 2. Results and Discussion

The spectral energy distribution of the particles in the beam is the most complete characteristic of an X-ray source. However, the radiation source is often characterized by only one parameter: tube voltage, HVL, or effective/average energy of the spectrum, which individually do not completely describe the properties of the beam. Due to the variety of medical and laboratory X-ray machines, as well as the many promising NP compositions, a quite simple principle to assess the efficacy of their combinations in terms of the absorbed dose enhancement is desirable. In this work, the selection of the optimal irradiation conditions for high-*Z* NPs of various compositions was performed for the clinically relevant energy range of kilovoltage X-ray (30–300 keV) [42]. Although the tube voltage is the most used X-ray characteristic, the lack of an obvious relationship between the voltage and DEF values does not allow for the use of tube voltage as the only parameter determining the properties of the X-ray beam. Therefore, the effective energy of the spectrum was chosen as another defining parameter of the beam, similarly to [30,43]. The average beam energy can be used instead of the E_eff_; however, its experimental determination is much more difficult.

**Figure 1:**
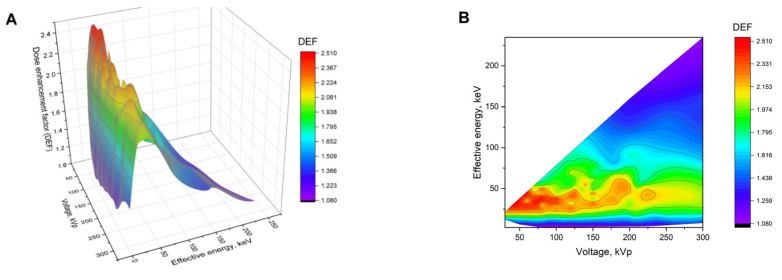

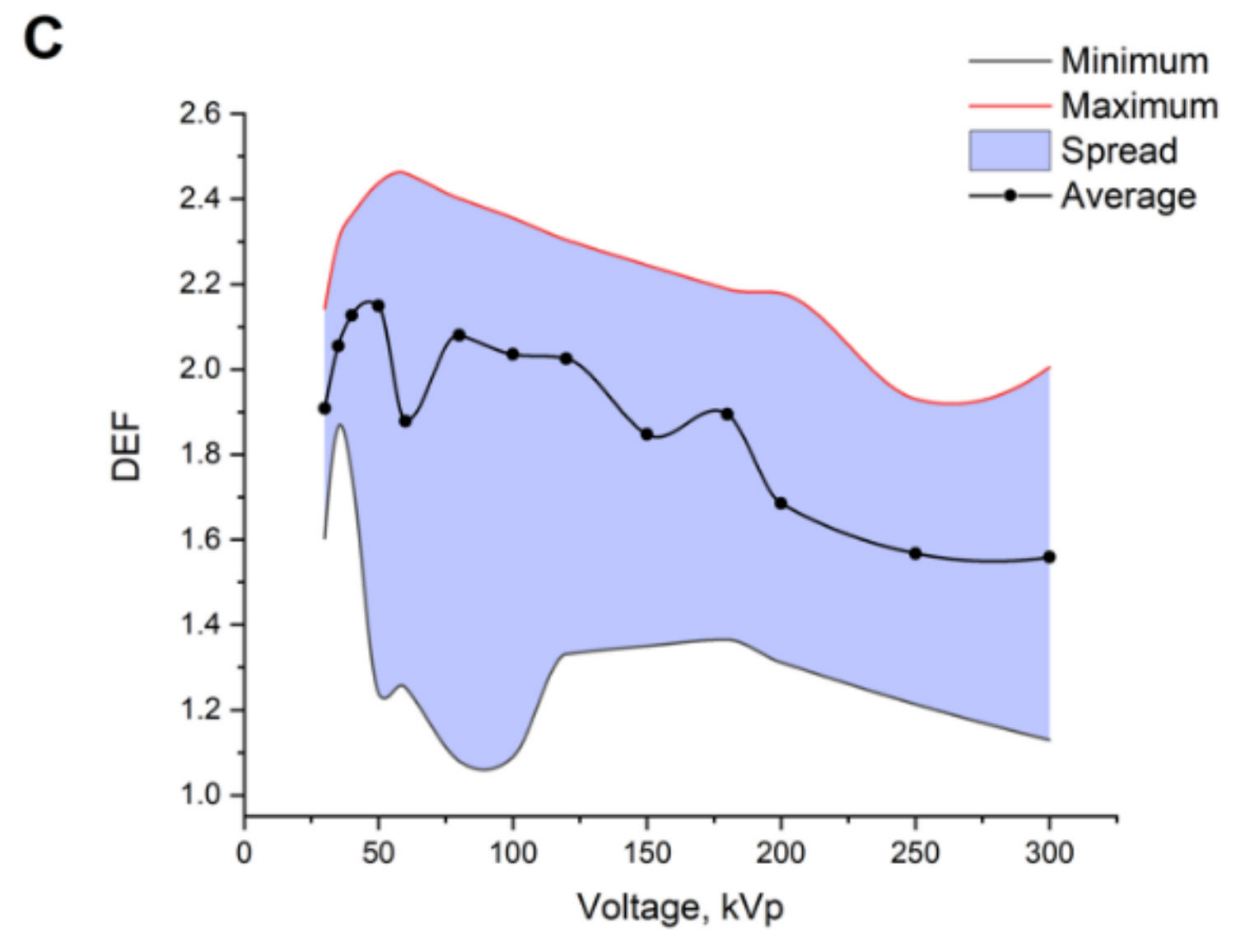
(**A**,**B**) Dependences of the dose enhancement factor (DEF) on the X-ray tube voltage and effective energy of the spectra for the Au NP concentration of 10 mg/mL: (**A**) 3D plot and (**B**) view from above. (**C**) The spread of DEF values at fixed voltages and various filtration parameters.

Figure 1A shows the dependence of DEF on the tube voltage and the effective energy of various spectra, calculated for an Au NP concentration of 10 mg/mL. The maximum DEF for the studied set of X-ray spectra was 2.46 (U = 60 kVp, E_eff_ = 44.8 keV). A more than 140% increase in the absorbed dose (DEF ≥ 2.4) was obtained for approximately 5% of the spectra. An increase in the absorbed dose of more than 50% (DEF ≥ 1.5) was noted for about 83% of the spectra, while 7% of them corresponded to a <30% dose enhancement. The graph is characterized by a pronounced region with the highest DEF values (U = 35–120 kVp; E_eff_ = 24–48 keV; highlighted in red), which is shown more clearly in Figure 1B. In terms of the absorbed dose enhancement, this region could be considered the most beneficial for the use of GNPs. It corresponds to a more than 130% increase in the absorbed dose, and the fraction of spectra with such an effect was about 18% of the studied set. The results obtained are in good agreement with previous works [25,43,44], where a greater GNPs radiosensitization was observed at lower X-ray tube voltages.

The tube voltage determines only the maximum energy of the photons in the spectrum, while their energy distribution depends on the construction of the X-ray source, including the filtration system used. Thus, the same voltage value can correspond to the various X-ray spectra, and hence to the different DEFs. The spread of DEF values at fixed voltages from 30 to 300 kVp is presented in Figure 1C. For example, at a 100 kVp voltage, the lowest DEF was 1.09, the highest one was 2.36, and the average value turned out to be 2.04. In addition, the close effective energies could correspond to the X-ray spectra generated under completely different conditions. For example, 13 spectra from the studied set with voltages from 50 to 250 kVp were characterized by the effective energy of ≈24 keV. The spread of DEF values for them ranged from 1.93 to 2.34, and the average value was 2.27.

**Figure 2:**
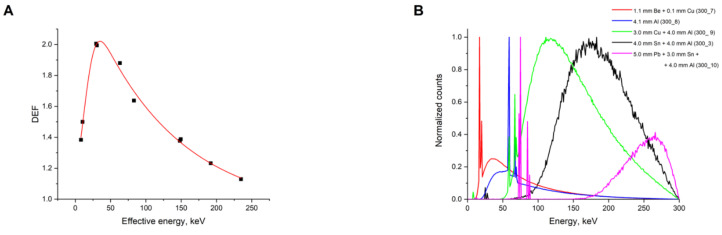

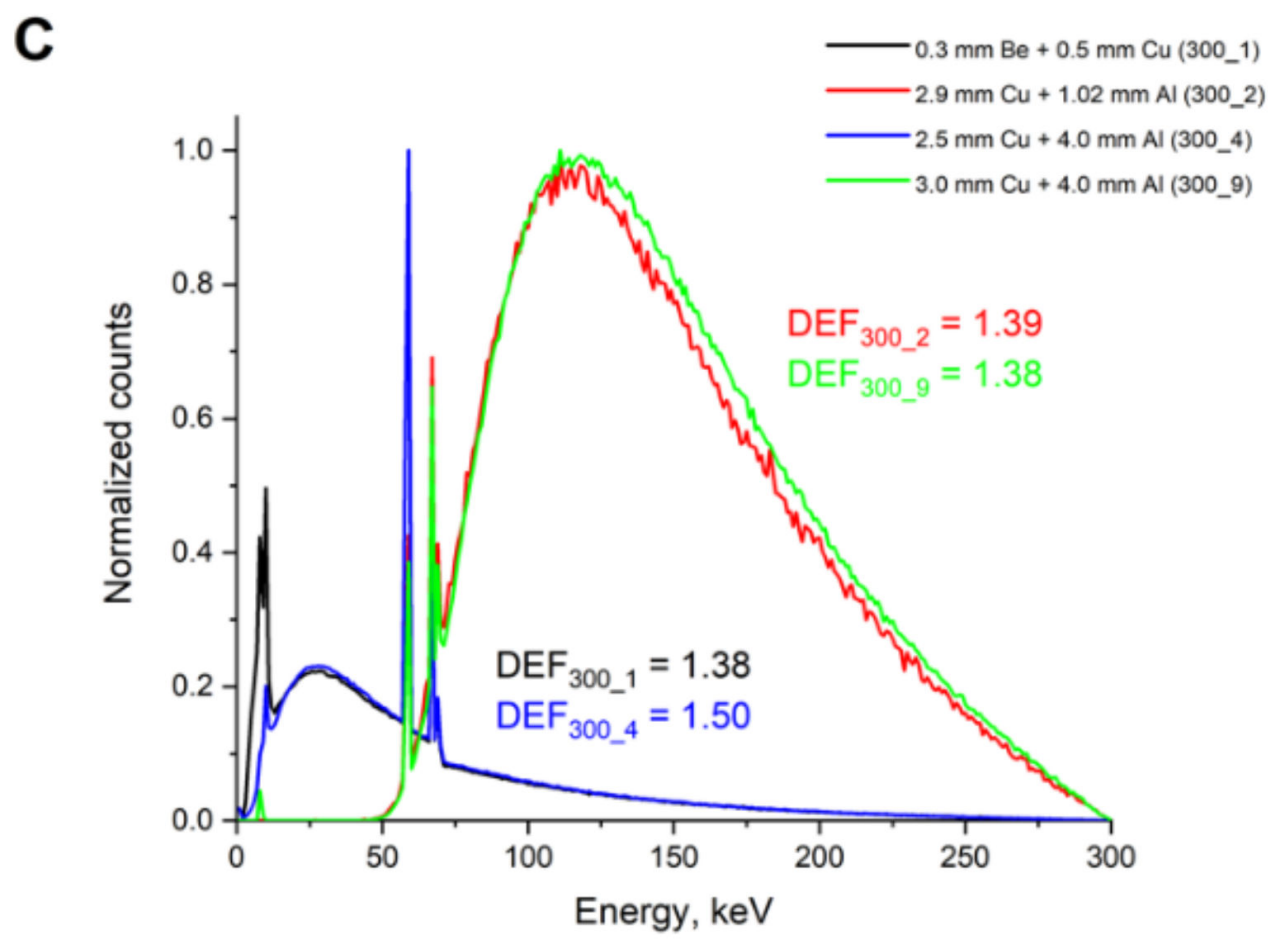
(**A**) Dependence of DEF on the effective energy of the spectra generated at a fixed 300 kVp voltage. (**B**) Examples of X-ray spectra calculated using the Monte-Carlo method at a fixed 300 kVp voltage and various filters. (**C**) Comparison of DEF values for spectra of different shapes.

As an example, Figure 2A shows the dependence of DEF on the effective energy of the X-ray spectra produced using various filters at a fixed voltage of 300 kVp. The parameters of the investigated radiation sources and of the generated spectra, as well as the corresponding DEFs, are presented in Table 1. The most significant differences in the simulated X-ray spectra are shown in Figure 2B. The shape of the obtained DEF vs. E_eff_ dependences is generally similar for the studied spectra with voltages above ≈100 kVp. For these cases, the peak on the curves lies in 20–80 keV range, but in some cases, it may be noticeably less pronounced. It is noteworthy that close DEF values can correspond to completely different spectra (e.g., spectra 300_1 (5) and 300_2 (73) in Figure 2C). At the same time, both differing (e.g., spectra 300_1 (5) and 300_4 (119)) and nigh DEF values (e.g., spectra 300_2 (73) and 300_9 (254)) can be observed for the close shape spectra.

**Figure 3:**
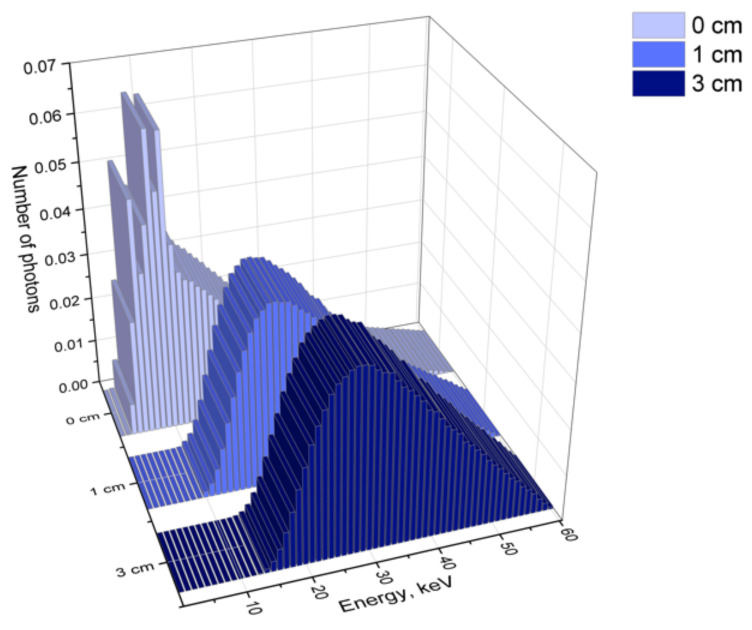
Photon energy distributions in a 60 kVp spectrum (60_7 (152)) at different depths in the water phantom calculated by the Monte-Carlo method. Anode—W, filtration–0.4 mm Be.

Kilovoltage X-rays are usually used for the treatment of neoplasms with a localization depth up to 3–5 cm [42]. It is important to note that when passing through the matter, the photon beam undergoes attenuation, the degree of which depends on the photon energy and the properties of the irradiated substance. The low-energy component of the X-ray spectrum undergoes the most significant changes. The corresponding changes in the 60 kVp spectrum (W, 0.4 mm Be) caused by the passage of the beam through the water phantom calculated by the Monte-Carlo method are shown in Figure 3 as an example. Thus, the X-ray spectrum at a certain depth can differ significantly from the initial one. This circumstance must be taken into account when comparing the efficacy of NPs radiosensitization in the cases where biological samples are placed in a phantom or under solid water during irradiation [45,46], as well as in open irradiation [34]. It is also of great importance for planning clinical exposure in the presence of nanoradiosensitizers.

**Figure 4:**
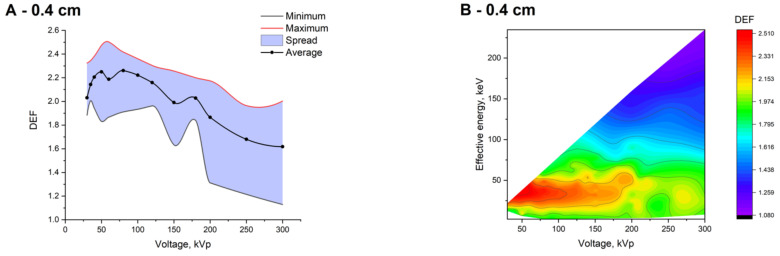

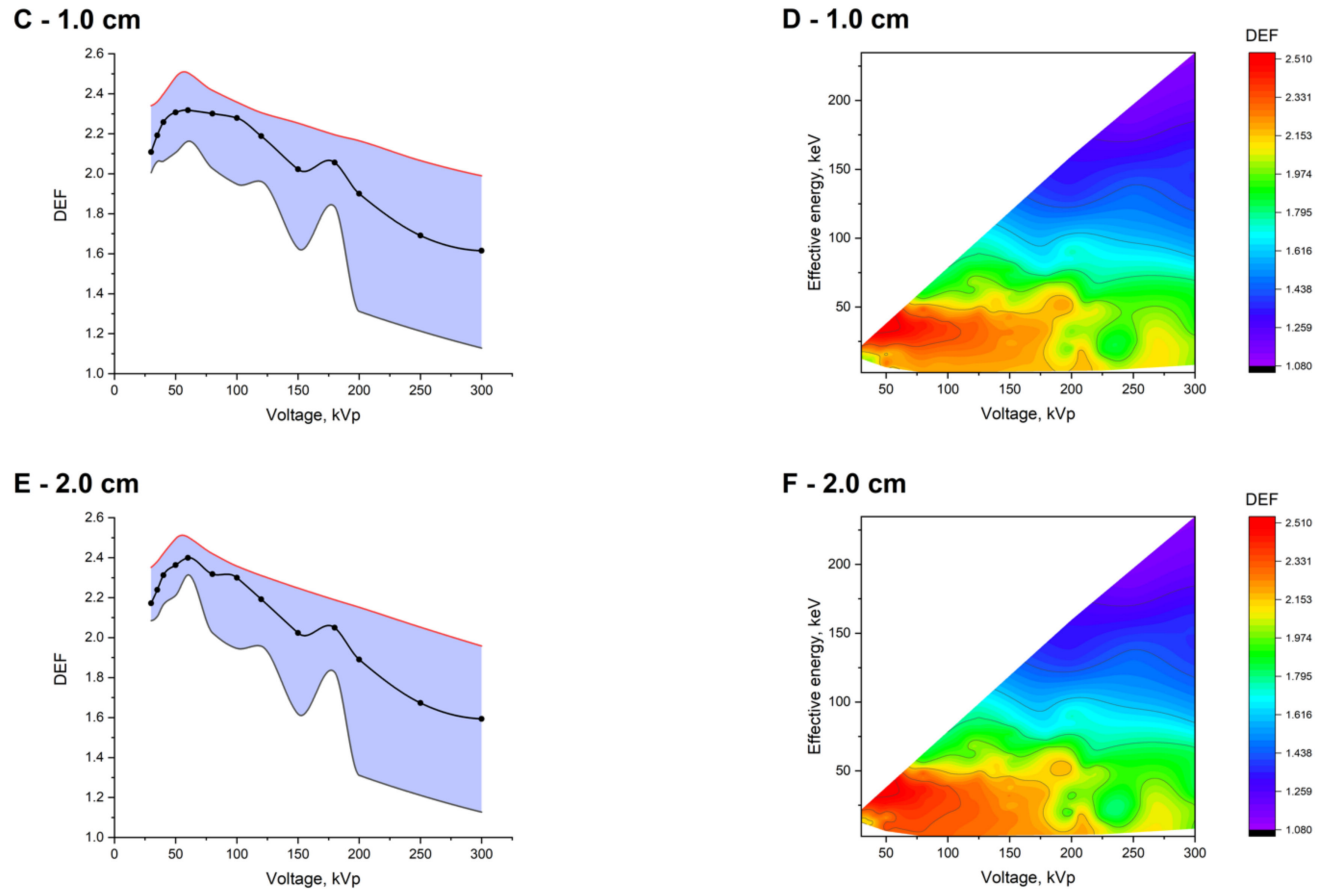
Spreads of DEF values at fixed tube voltages (**A**,**C**,**E**), and dependencies of DEF on the voltage and effective energy of the studied spectra (**B**,**D**,**F**) at an Au concentration of 10 mg/mL at different depths in the water phantom: 0.4 cm (**A**,**B**), 1.0 cm (**C**,**D**), and 2.0 cm (**E**,**F**). Heat maps here (**B**,**D**,**F**) and below show the effective energies of initial spectra.

Figure 4 shows the spreads of DEF values at fixed tube voltages and the dependencies of DEF on the tube voltage, as well as the effective energy of the tested spectra at different depths in a water phantom at an Au concentration of 10 mg/mL. Even at a 0.4 cm depth, the spread of DEF values significantly reduced at the voltages up to about 200 kVp. At the same time, the number of spectra for which the absorbed dose enhancement was less than 30% decreased by about six times (to 1%). For example, the minimum DEF increased from 1.09 to 1.93 at 100 kVp voltage. An increase in the absorbed dose caused by a change in the initial spectrum according to depth and providing a noticeable increase in the average DEF values (from 2% to 28% depending on the voltage) was noted for a significant part of the studied X-ray spectra. At a depth of 0.4 cm, the DEF exceeded 1.5 what was observed for 96% of the spectra (versus 83% at the surface). At the same time, the maximum DEF increased insignificantly (from 2.46 to 2.50). The number of spectra corresponded to the absorbed dose enhancement of more than 140% also increased insignificantly, from 5% to 7%. Thus, an increase in DEF with depth was primarily observed for the spectra that showed a small or moderate dose enhancement on the surface. The spread of DEF values did not change as significantly at higher voltages. Moreover, a decrease in DEF with an increasing depth was noted for some X-ray spectra generated at voltages above 180 kVp. At the same time, an increase in the size of the high-DEF region was observed with an increasing depth (highlighted in red in Figure 4B,D,F). The expansion of this region mainly took place towards low E_eff_ and high voltage values. With a further increase in depth from 2 to 5 cm, the changes in DEF were not significant (data not shown).

**Figure 5:**
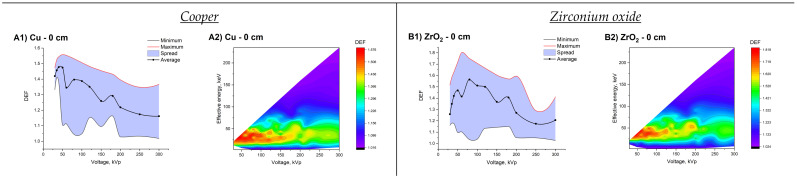

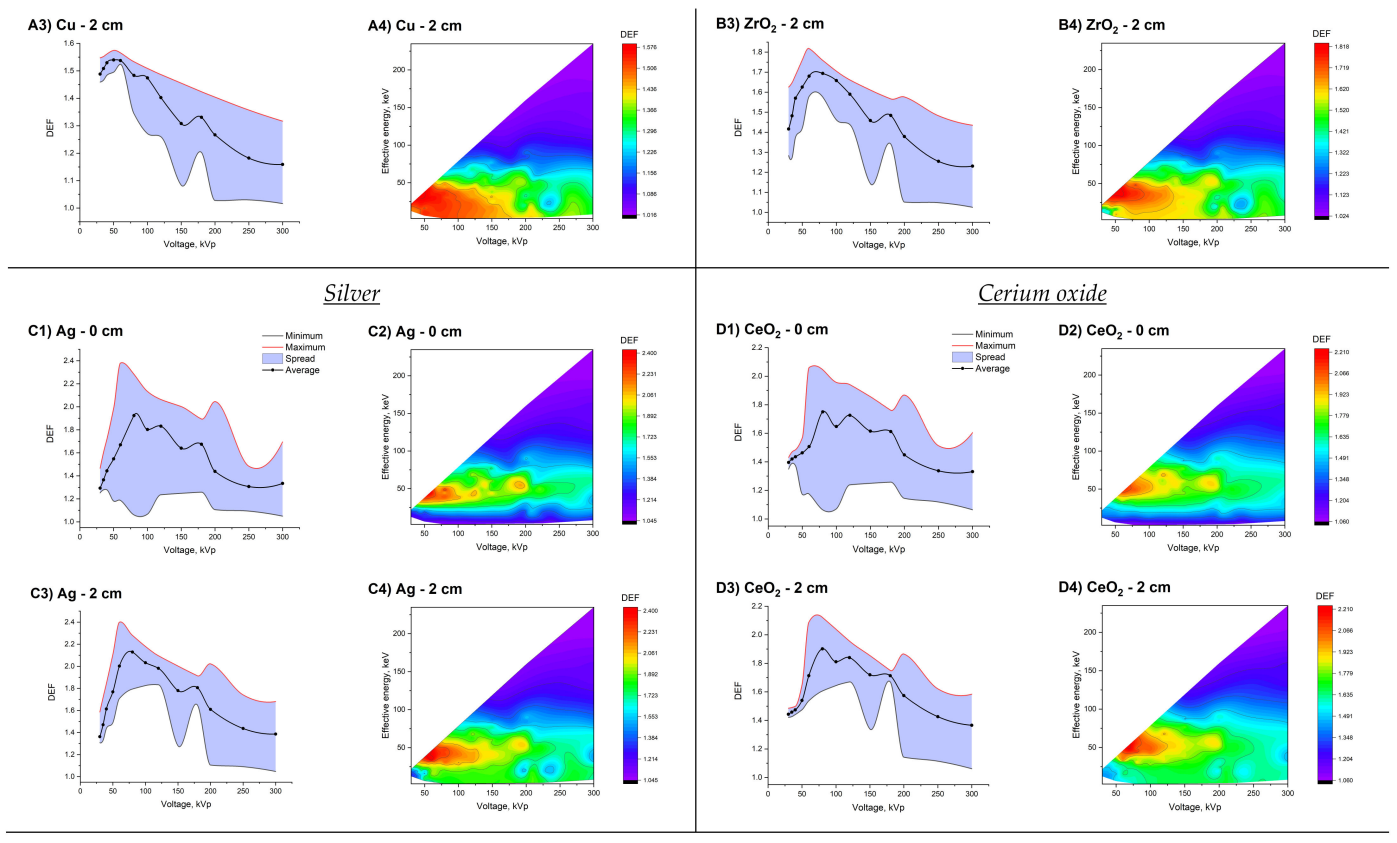

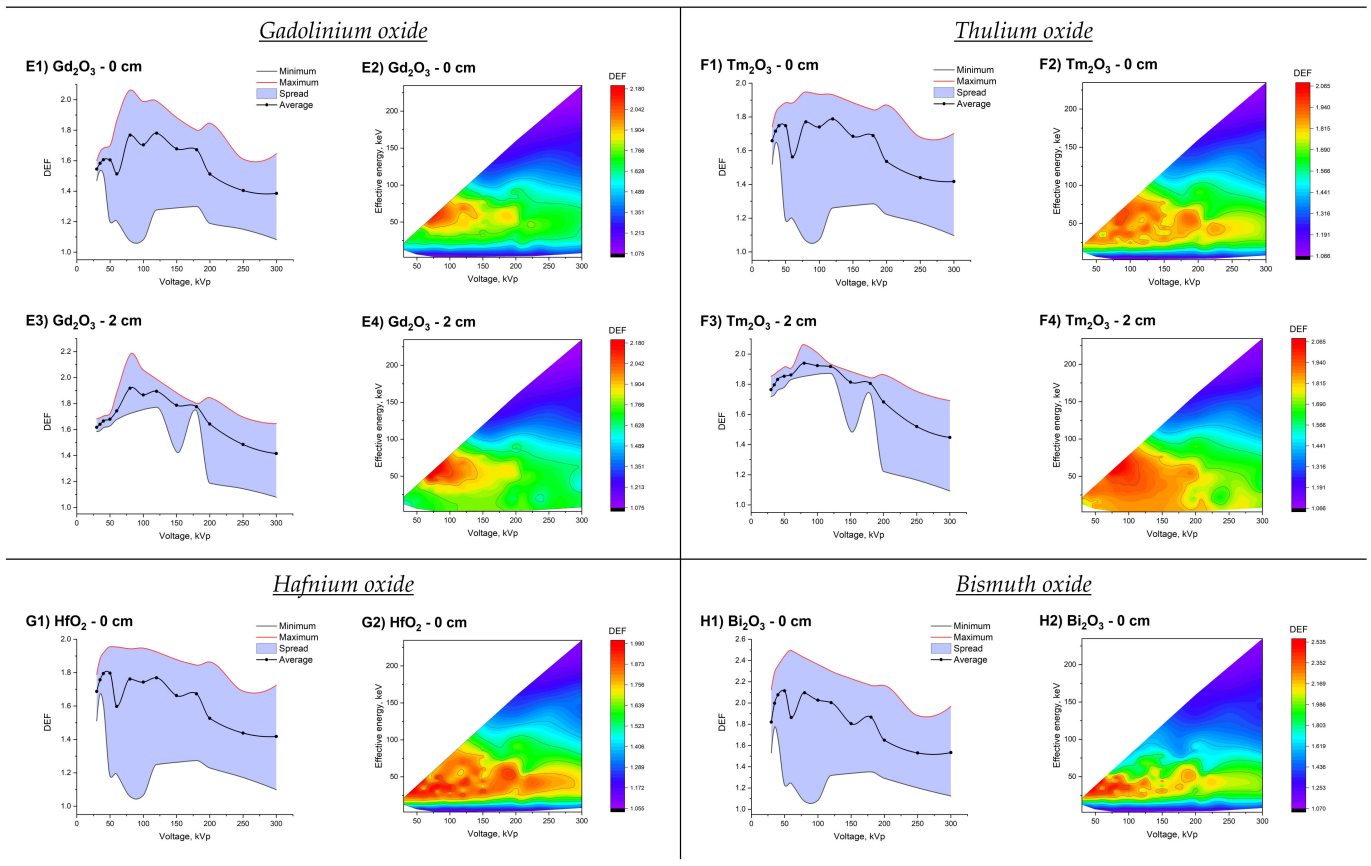

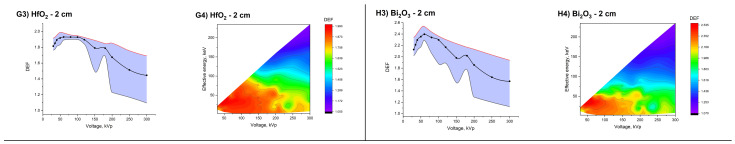
Spreads of DEF values as a function of X-ray tube voltage and dependence of DEF on the tube voltage and effective energy of spectra at different depths in a water phantom for 10 mg/mL of Cu (section (**A**)), ZrO_2_ (section (**B**)) Ag (section (**C**)), CeO_2_ (section (**D**)), Gd_2_O_3_ (section (**E**)), Tm_2_O_3_ (section (**F**)), HfO_2_ (section (**G**)), and Bi_2_O_3_ (section (**H**)).

Even though the set of NPs studied in combination with radiation are growing steadily, significant differences in experimental conditions and particle design do not allow for unambiguously judging which composition of NPs will be the most effective. By analogy with previous works [29,34,41], we compared 11 metal and metal oxide NPs in terms of their ability to enhance the absorbed dose. The results of their comparison for different X-ray spectra and localization depths are presented in Table 2, Table 3 and Table 4, as well as in Figure 5. As HfO_2_ and Ta_2_O_5_, as well as Pt and Au, showed almost the same DEF values, only one material from each of these pairs is presented in the tables and figures (HfO_2_ and Au, respectively).

The largest increase in the absorbed dose among the entire set of studied materials was noted for Bi_2_O_3_ NPs (DEF = 2.49–2.53 depending on depth). The maximum DEF for Au NPs was only 1% less. At the same time, Au exceeded Bi_2_O_3_ in terms of the average DEF (1.95–2.17 vs. 1.91–2.15 depending on depth) and in the number of spectra with a high effect (DEF >2; 59–142 vs. 58–131 depending on depth). More than 100% increase in the absorbed dose (DEF ≥ 2) at the studied concentration was noted for NPs of Tm_2_O_3_, Gd_2_O_3_, CeO_2_, and Ag. However, among them, only for Ag NPs was such an effect observed for a significant (>10%) number of X-ray spectra from the studied set. For example, in the case of Tm_2_O_3_ NPs, a two-fold absorbed dose enhancement was found for only 2 spectra at a depth of 0.4 to 2.0 cm. In addition, the maximum DEF for Ag NPs (DEF = 2.38–2.40 depending on depth) significantly exceeded the corresponding ones for materials with a higher *Z*, conceding only to NPs of Bi_2_O_3_ and Au. The smallest DEF values were demonstrated by NPs of Cu and ZrO_2_. The maximum absorbed dose enhancement was 56–58% in the case of Cu and 80–82% for ZrO_2_. The average DEFs for these materials at different depths were 1.36–1.43 and 1.39–1.53, respectively. The obtained DEF values were notably conceded ones for a significant part of previously studied set of magnetic NPs taken at the same concentration [41].

In general, the change in the X-ray spectral composition with depth for all of the studied high-*Z* NPs, considering certain differences for individual spectra and materials, coincided with the above-described trend for Au NPs. An increase in depth led to an increase in DEF and an expansion of the high-DEF region. At the same time, the sizes and characteristics of the high-DEF regions for various NPs can differ significantly. The largest size of such a region (in coordinates U, E_eff_) occurs for HfO_2_ and Ta_2_O_5_. The DEF values in the range from 1.8 to 2.0 were observed in combination with 164 of 237 of the studied spectra (69%). Thus, NPs of HfO_2_ and Ta_2_O_5_ can be the most versatile.

**Figure 6:**
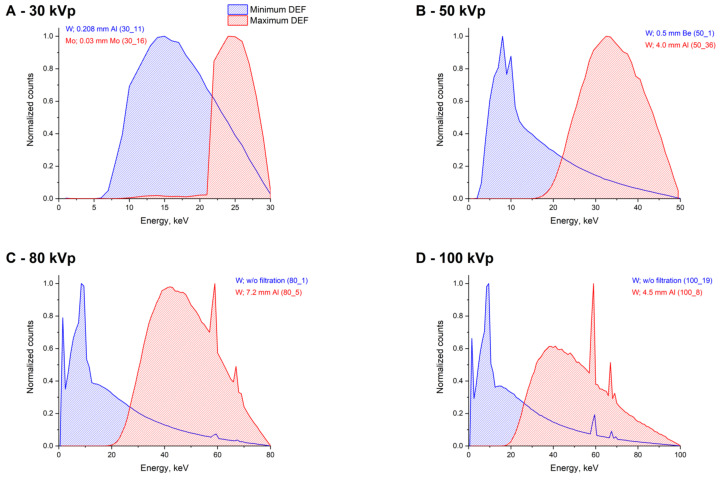

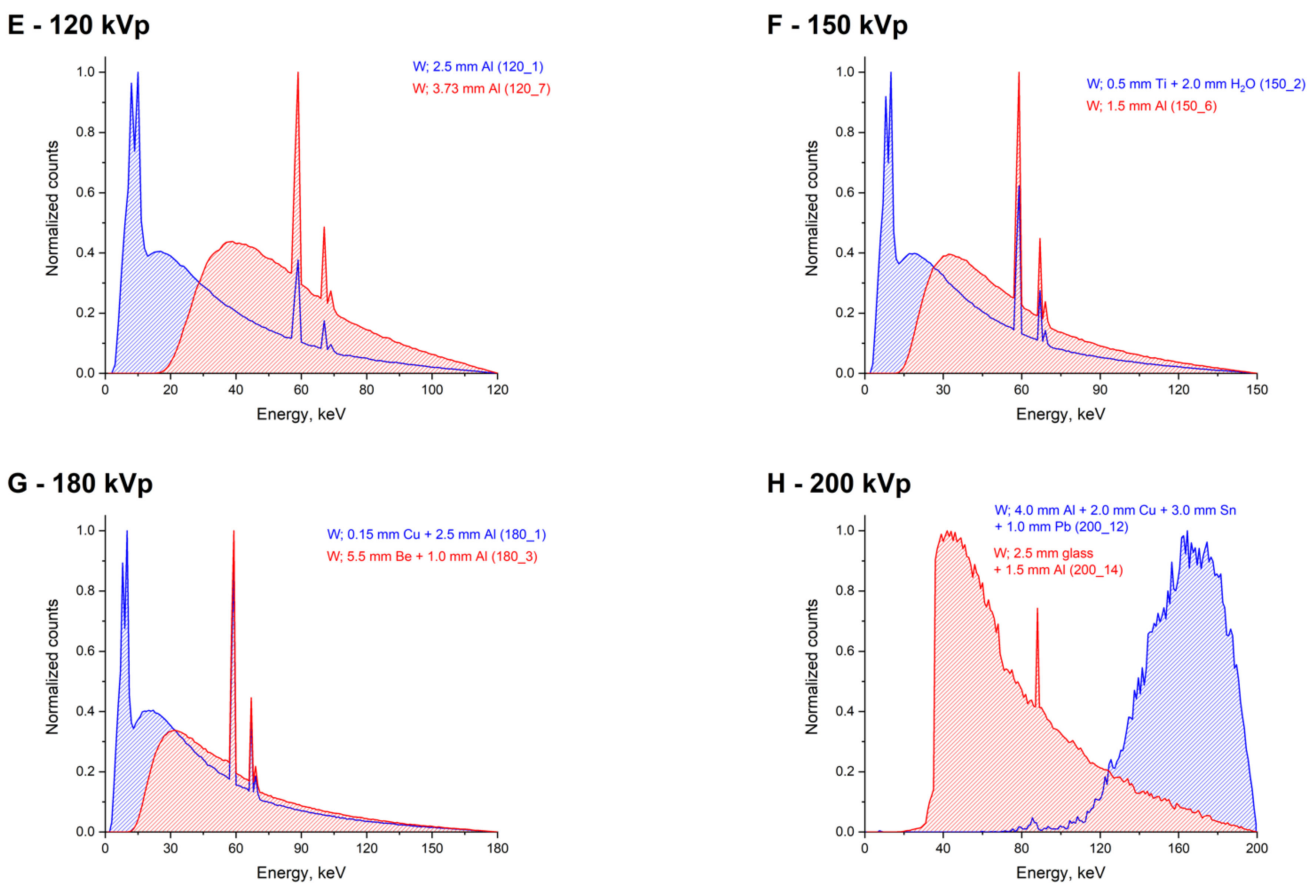
The examples of the X-ray spectra that correspond to the minimum and maximum DEF values for most of the studied NPs. The numbers of spectrum are shown in the brackets according to the Supporting Information.

The spectra, which showed a slight absorbed dose enhancement in the case of Au, also demonstrated relatively low values for other materials. An increase in depth in general also led to significant increase in DEF values. In terms of DEF, the worst (considering depth caused changes) were the spectra formed at voltages above 100 kVp and containing a significant high-energy component with the absence of photons with energies below 50 keV. For example, no DEF values below two were observed at voltages below 100 kV for Au at a depth of 1 cm. The maximum DEFs were observed for the spectra containing a large number of photons with energies matching the maximum difference in the mass energy-absorption coefficients of the given material and water. However, such spectra did not show a noticeable increase in DEF as the depth increased. The examples of the corresponding spectra with minimum and maximum DEF values on the surface in the voltage range 30–200 kVp are shown in Figure 6.

As noted earlier [26], a higher DEF does not always correspond to a higher *Z* for polychromatic X-rays. The most remarkable in this regard was Ag. Although it has a much lower atomic number than CeO_2_, Gd_2_O_3_, Tm_2_O_3_, and HfO_2_, in some cases, Ag was not inferior to these materials according to the DEF value. For example, for 26–32% (depending on the depth) of the studied spectra, Ag showed a greater dose enhancement compared with hafnium oxide. In addition, the higher DEF values compared with cerium oxide for almost all of the spectra below 60 kVp were found for Cu. For such spectra, in several cases, Cu turned out to be more efficient than Ag. However, when the low-energy component of the spectrum attenuated as the depth increased, the dominance of silver was noted. This effect was practically absent for the Cu–CeO_2_ pair. For the studied set of X-ray spectra, the highest efficiency was demonstrated by Au. Only in 13–19% of cases it was conceded to Bi_2_O_3_ and in 1–2% of cases to Tm_2_O_3_, Gd_2_O_3_, CeO_2_, and Ag. Compared with the rest of the NP compositions, Au was the absolute leader.

**Figure 7:**
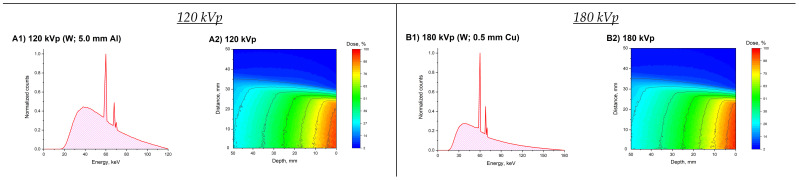

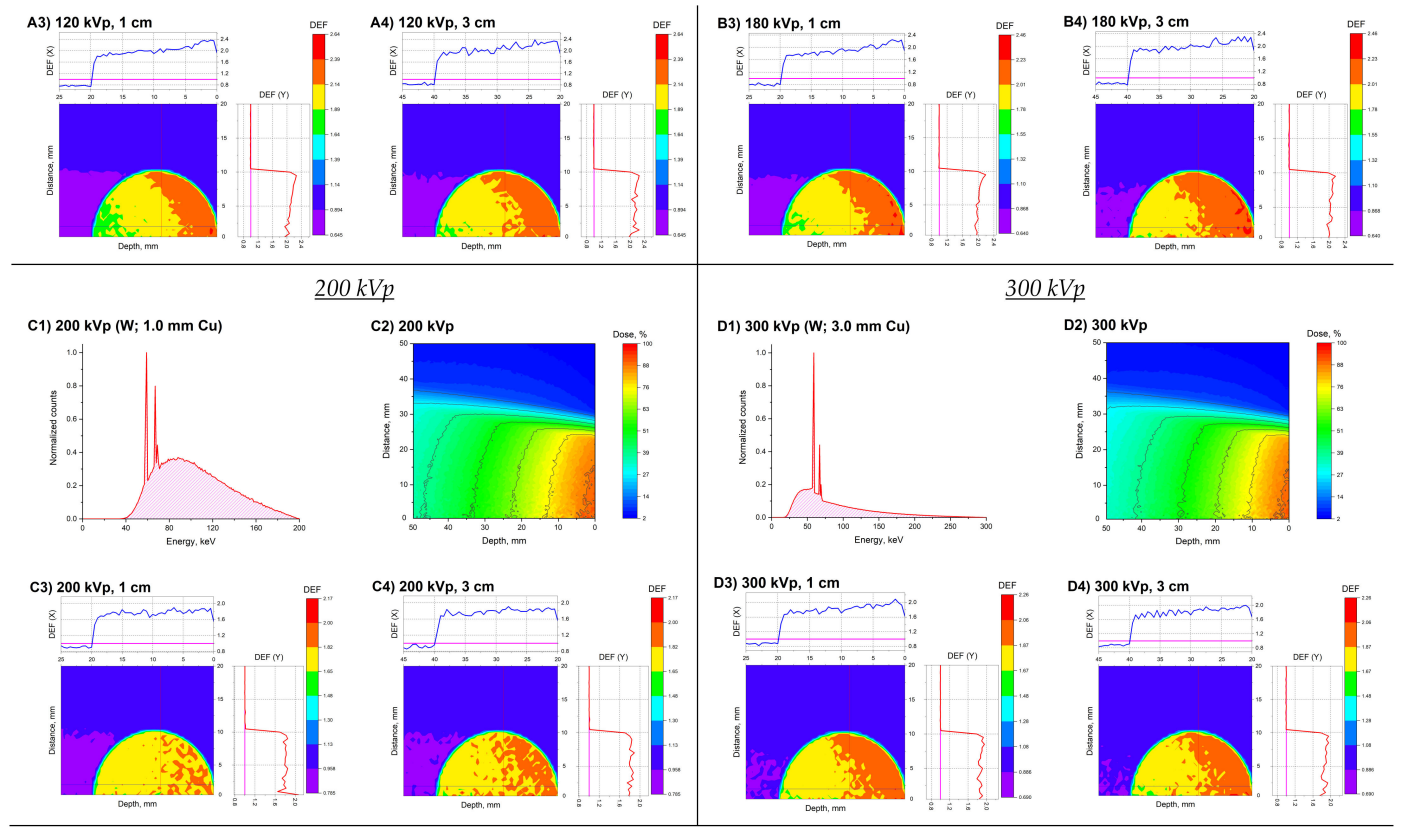
Block (**A**)—120 kVp; block (**B**)—180 kVp; block (**C**)—200 kVp; block (**D**)—300 kVp. In each block, the figures numbered as 1 show simulated X-ray spectra (A1, B1, C1, and D1). Figures at numbered as 2 show the calculated depth dose distributions. The spatial distributions of DEF within the model tumor are demonstrated by numbers 3 and 4. Figures numbered by 3 correspond to model tumors centered at a depth of 1 cm, numbered by 4–at a depth of 3 cm, respectively.

Furthermore, irradiation of a 2 cm in diameter spherical model tumor located at depths of 1 or 3 cm in a water phantom was simulated using the Monte-Carlo method. The conventional scenario of single-field exposure was considered. As among the studied materials with high-Z NPs gold showed the greatest efficacy, the presence of 10 mg/mL Au NPs in the model tumor was simulated. The calculated spectra generated using clinically relevant voltages of 120, 180, 200, and 300 kVp and the corresponding depth dose distributions, as well as spatial distributions of DEF within the model tumor, are shown in Figure 7. For the considered spectra, the DEF values were significantly different. The lowest DEF values (up to 2.17) were observed for the 200 kVp spectrum, while for the spectra formed at voltages of 120 and 180 kVp, the DEF values were up to 2.64 and 2.46, respectively. This result is in good agreement with the above-described data, as the fraction of photons with energies less than 50 keV in the 200 kVp spectrum is significantly lower than in the others. At the same time, a decrease of up to about 20% in the dose behind the model tumor was observed for the 120, 180, and 200 kVp spectra. For the 300 kVp spectrum, the weakest dosage decrease was found.

The spatial distributions of the DEF values within the model tumor were also significantly different. For example, a spread of DEF values from 1.64 near the lower boundary of the tumor to 2.64 in its upper part was observed inside a sphere located at a depth of 1 cm for a 120 kVp spectrum. An increase in the location depth of the model tumor in the water phantom led to notable increase in the uniformity of DEF spatial distribution in all cases except the 300 kVp spectrum. However, in all cases, the uniformity of the dose coverage of the model tumor was found to be unsatisfactory. Thus, the previously shown scheme of multifield irradiation for a similar model tumor in the presence of GNPs looks more promising [32].

## 3. Materials and Methods

### 3.1. Analytical Calculation of Dose Enhancement Factor

To assess the efficacy of the studied NPs with different compositions, the dose enhancement factor (DEF) values were calculated. DEF is defined as the ratio of the dose absorbed in the volume of interest in the presence of NPs (*D*_2_) to the dose absorbed in the same volume in their absence (*D*_1_):

(1) $$DEF=\frac{D_{2}}{D_{1}}.$$

If the absorbed dose $D_{1}$ in substance 1 is known, then, under conditions of electronic equilibrium, the absorbed dose $D_{2}$ in substance 2 at the same point of the irradiation field can be calculated using Equation (2).

(2) $$D_{2}=\frac{{\left(\mu /\rho \right)}_{2}}{{\left(\mu /\rho \right)}_{1}}D_{1},$$

where ${\left(\mu /\rho \right)}_{i}$ is the photon mass energy-absorption coefficient of the *i*-th substance. In the case of monochromatic radiation and a small volume of interest, the conditions of electronic equilibrium make it possible to determine the absorbed dose with a ≤10% error. In the case of a polychromatic source, Equation (2) takes the following form.

(3) $$D_{2}=\frac{\int_{0}^{E_{max}}\varphi \left(E\right)E{\left(\frac{\mu \left(E\right)}{\rho }\right)}_{2}dE}{\int_{0}^{E_{max}}\varphi \left(E\right)E{\left(\frac{\mu \left(E\right)}{\rho }\right)}_{1}dE}D_{1}=\frac{\bar{{\left(\frac{\mu }{\rho }\right)}_{2}}}{\bar{{\left(\frac{\mu }{\rho }\right)}_{1}}}D_{1}=\bar{{\left(\frac{\mu }{\rho }\right)}_{1}^{2}}D_{1},$$

In Equation (3), $\varphi \left(E\right)dE$ stands for the flux of photons, whose energy lies in the interval (E, E+dE). Thus, we obtain the following expression for DEF.

(4) $$DEF=\bar{{\left(\frac{\mu }{\rho }\right)}_{1}^{2}}$$

For a substance composed of several chemical elements, the mass absorption coefficient can be calculated by the equation below:

(5) $$\frac{\mu_{en}}{\rho }=\underset{i}{\sum }w_{i}{\left(\frac{\mu_{en}}{\rho }\right)}_{i},$$

where ${\left(\frac{\mu_{en}}{\rho }\right)}_{i}$ is the mass energy-absorption coefficient of the *i*-th element in the substance and $w_{i}$ is the weight fraction of this element.

The characteristics of the substances forming the studied high-*Z* NPs are presented in Table 5. As the NPs with a *Z* below 28 (nickel) investigated in the previous work did not show high dose enhancement [26], here, we examined NPs based on metals with a *Z* from 29 (copper) to 83 (bismuth). The NP concentration in water was equal to 10 mg/mL, according to [47]. Concentrations of this order are widely studied in theoretical works [32,48] and are also relevant to in vitro and in vivo conditions [49]. The values of the photon mass energy-absorption coefficients were obtained using the XMuDat software [50], based on the data by Hubell J.H. and Seltzer S.M. [51]. The effective atomic numbers, $Z_{eff}$, for multi-elemental NPs were calculated using the Mayneord equation:

(6) $$Z_{eff}=\sqrt[{2.94}]{\overset{n}{\underset{i=1}{\sum }}a_{i}Z_{i}^{2.94}}$$

where $a_{i}$ is s the relative electron fraction of the *i*-th element.

### 3.2. Monte-Carlo Simulation of X-ray Spectra

**Figure 8:**
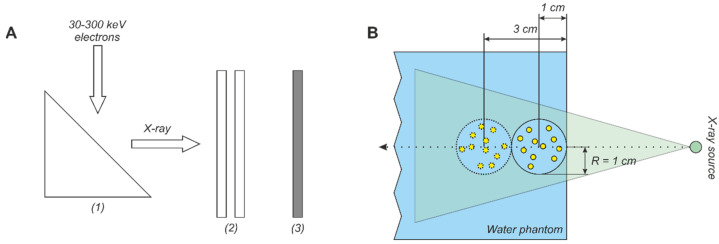
(**A**) Schematic presentation of the geometry of the X-ray spectra simulation: (1) anode, (2) filtration system, (3) X-ray detector. (**B**) Diagram explaining the simulation of the X-ray irradiation of the model tumor in the presence of gold NPs (GNPs).

The X-ray spectra were simulated by the Monte-Carlo method using the Geant4 software (PhysicsList, Livermore; energy cut − 0.01 mm) [52]. The geometry of the used model is shown schematically in Figure 8A. A 1 mm×2 mm rectangular electron beam fell on anode (*1*) located at an angle of 20 degrees to the beam axis. For the analytical calculation of DEF for the studied NP compositions we used 237 various X-ray spectra. The electron beam energy was varied from 30 to 300 keV. Rhodium and molybdenum (for about 6% and 12% of the studied spectra, respectively) were used as an anode material, besides the most common tungsten (≈82% of the spectra). The filtration system (*2*) was simulated by layers of Al, Cu, Pd, Mo, Rh, Ba, Sb, V, Mn, Fe, Ni, Zr, Sn, Pb, and H_2_O of different thicknesses in various combinations. The beryllium window of a 2-mm thickness was used as the default. The X-ray radiation spectrum generated in the anode was recorded with a detector (*3*), fixed at 30 cm from the filtration system. For each combination of parameters, the passage of 2 × 10^8^ primary photons was simulated. The characteristics of X-ray tubes and generated spectra used in the analytical calculation of DEF are presented in the Supplementary Materials (Tables S1–S29).

The change in the initial spectrum $\varphi \left(E\right)$ as the photon beam passes through the water layer of thickness d (up to 5 cm) was calculated using the equation below:

(7) $$\varphi \left(E,d\right)\;=\;\varphi \left(E\right)\;\times \;\exp\left(-\mu_{en(H_{2}O)}\left(E\right)\;\times \;d\right)$$

The effective energy of an X-ray spectrum $\varphi \left(E\right)$ is defined as the energy of such a monoenergetic beam, for which the half-value layer (HVL) in aluminum is equal to the HVL for a given spectrum. For the photon beams with a continuous energy spectrum, the HVL can be found according to the following equation:

(8) $$\int_{0}^{E_{\gamma }^{max}}E_{\gamma }\frac{dN_{\gamma }}{dE_{\gamma }}e^{-\mu \left(E_{\gamma }\right)HVL}dE_{\gamma }=\frac{1}{2}\int_{0}^{E_{\gamma }^{max}}E_{\gamma }\frac{dN_{\gamma }}{dE_{\gamma }}dE_{\gamma }$$

where $E_{\gamma }^{max}$ is the maximum photon energy in the spectrum, $\frac{dN_{\gamma }}{dE_{\gamma }}dE_{\gamma }$ is the fraction of photons with energy from $E_{\gamma }$ to $E_{\gamma }+dE_{\gamma }$, and $\mu \left(E_{\gamma }\right)$ is the energy dependence of Al attenuation coefficient. For the investigated X-ray beams, the HVL was determined from the numerical solution of the equation

(9) $$\underset{i}{\sum }E_{i}N\left(E_{i}\right)e^{-\mu \left(E_{i}\right)HVL}=\frac{1}{2}\underset{i}{\sum }E_{i}N\left(E_{i}\right)$$

where $N\left(E_{i}\right)$ is the number of photons with energy in the $\left(E_{i}-\Delta E,E_{i}+\Delta E\right)$ interval and *i* is the number of intervals into which the spectrum is subdivided (ΔE=0.5 kev). The effective energy is related to the HVL value as follows:

(10) HVL=ln2μEγ

### 3.3. Model Tumor Simulation

To study the impact of the X-ray spectral properties on the uniformity of the dose coverage of the irradiated target in the presence of the high-*Z* NPs, a spherical model tumor with a radius R=2 cm was simulated. The geometry and size of the model were chosen according to [32]. The model tumor was placed in a 20 cm×20 cm×20 cm water phantom and was centered on the axis of the photon beam at a depth of 1 or 3 cm (Figure 8B). Water was also used as a material for the model tumor. The presence of 10 mg/mL NPs was set by changing the mass composition of the model tumor, assuming a uniform distribution of the NPs. The absorbed dose was measured in a cylindrical grid with a step of 1 mm along both the radius and depth directions.

In this Monte-Carlo simulation, we used four additional X-ray spectra. The characteristics of the used radiation sources were the following: 120 kVp (filtration: 0.5 mm Al + 0.1 mm Cu; HVL: 5 mm Al), 180 kVp (1.5 mm Al + 0.15 mm Cu; HVL: 0.5 mm Cu), 200 kVp (1.0 mm Al + 0.5 mm Cu; HVL: 1 mm Cu), and 300 kVp (1.5 mm Al + 0.25 mm Cu; HVL: 3 mm Cu). As low-energy (≤50 kVp) X-rays are usually used for the treatment surface foci of the disease [42], spectra generated at voltages (U) above 100 kVp were chosen for the simulation. In all these four cases, filtration using 3 mm of beryllium was simulated by default. Tungsten was used as the anode material. The spectra were modeled in accordance with the above procedure (Section 3.2). The obtained spectra correspond, by their HVL values, to the radiation of clinical devices, for example, Xstrahl-150^®^, Xstrahl-200^®^, and Xstrahl-300^®^ [53].

## 4. Conclusions

In the present work, we proposed a method for the analytical evaluation of the most efficient combinations of high-*Z* nanoradiosensitizers and kilovoltage X-rays in terms of the enhancement of the absorbed dose. Among the 11 investigated metal and metal oxide NPs (from Cu (*Z*_Cu_ = 29) to Bi_2_O_3_ (*Z*_Bi_ = 83)), gold was superior. In addition, a great potential for dose enhancement was noted for Pt, Bi_2_O_3_, and Ag NPs. The oxides of cerium, gadolinium, thulium, hafnium, and tantalum showed quite good effects. However, as the dependence of DEF on the spectral composition is very complex, it is necessary to consider each specific case separately. It was shown that there are X-ray sources that correspond to an extremely low increase in the absorbed dose for all of the studied NPs. Characteristic features of these spectra are the predominance of a high-energy component, as well as a low number of photons with energies less than ≈50 keV. It was also shown that a change in the spectral composition of kilovoltage X-rays caused by the photon beam attenuation when passing through the matter can significantly affect the DEF value. The largest increase in DEF as the depth increases was found for the spectra with quite small DEFs at the surface. For the spectra with high values of DEF at the surface, the growth was quite weak. A decrease in DEF while the depth increased was found for several spectra generated at voltages above 180 kVp. In addition to the X-rays spectral composition, the geometry and localization of the tumor can play an important role in the NPs radiosensitization efficacy. All of these findings indicate the important role of careful planning radiation exposure in the presence of high-*Z* NPs, and the necessity for the development of superficial X-ray techniques in parallel with the improvement of nanoradiosensitizers.

## Figures and Tables

**Table 1:** Characteristics of X-ray tubes and spectra generated at a fixed 300 kVp voltage and corresponding DEF values for the presence of Au NPs.

Spectrum Number	Anode Material	Filtration System	Average Energy, keV	Effective Energy, keV	DEF
300_1 (5)	W	0.3 mm Be + 0.5 mm Cu	62.24	7.98	1.38
300_2 (73)	W	2.9 mm Cu + 1.02 mm Al	144.93	149.34	1.39
300_3 (74)	W	4.0 mm Sn + 4.0 mm Al	186.84	191.80	1.23
300_4 (119)	W	2.5 mm Cu + 4.0 mm Al	66.94	10.25	1.50
300_5 (122)	W	6.5 mm Sn + 4.0 mm Al	75.09	30.59	1.99
300_6 (124)	W	4.1 mm Be + 0.4 mm Cu	102.77	83.06	1.64
300_7 (126)	W	1.1 mm Be + 0.1 mm Cu	72.25	29.56	2.01
300_8 (129)	W	4.1 mm Al	85.79	63.21	1.88
300_9 (254)	W	3.0 mm Cu + 4.0 mm Al	147.06	148.12	1.38
300_10 (260)	W	5.0 mm Pb + 3.0 mm Sn + 4.0 mm Al	235.62	234.76	1.13

**Table 2:** Minimum (DEF_min_), average (DEF_av_), and maximum (DEF_max_) DEF values for the high-*Z* NPs of various compositions at a different depths in water phantom.

	Cu	ZrO_2_	Ag	CeO_2_	Gd_2_O_3_	Tm_2_O_3_	HfO_2_	Bi_2_O_3_	Au
At the surface									
DEF_min_	1.02	1.03	1.05	1.06	1.07	1.07	1.06	1.08	1.08
DEF_av_	1.36	1.39	1.56	1.51	1.61	1.68	1.69	1.91	1.95
DEF_max_	1.56	1.80	2.38	2.07	2.06	1.95	1.96	2.49	2.46
At 0.4 cm depth									
DEF_min_	1.02	1.03	1.05	1.06	1.08	1.10	1.10	1.13	1.13
DEF_av_	1.42	1.46	1.64	1.57	1.68	1.77	1.79	2.05	2.09
DEF_max_	1.57	1.82	2.40	2.20	2.17	2.06	1.98	2.53	2.50
At 1.0 cm depth									
DEF_min_	1.02	1.03	1.05	1.06	1.08	1.09	1.10	1.12	1.13
DEF_av_	1.43	1.49	1.69	1.60	1.70	1.79	1.82	2.11	2.14
DEF_max_	1.57	1.82	2.40	2.21	2.18	2.06	1.98	2.53	2.51
At 2.0 cm depth									
DEF_min_	1.02	1.03	1.05	1.06	1.08	1.09	1.10	1.12	1.13
DEF_av_	1.43	1.53	1.74	1.62	1.72	1.81	1.83	2.15	2.17
DEF_max_	1.58	1.82	2.40	2.21	2.18	2.06	1.99	2.53	2.50

**Table 3:** Distribution of spectra by the DEF for NPs of different compositions at various depths.

	Cu	ZrO_2_	Ag	CeO_2_	Gd_2_O_3_	Tm_2_O_3_	HfO_2_	Bi_2_O_3_	Au
At the surface									
<1.2	53	50	18	15	8	12	13	4	4
1.2–1.4	55	71	75	48	32	28	27	35	34
1.4–1.6	129	73	48	98	60	10	11	4	3
1.6–1.8	-	42	37	46	98	110	104	33	18
1.8–2.0	-	1	30	25	37	77	82	58	54
2.0–2.2	-	-	24	5	2	-	-	45	65
>2.2	-	-	5	-	-	-	-	58	59
At 0.4 cm depth									
<1.2	21	13	8	4	4	3	3	1	1
1.2–1.4	45	82	44	11	5	6	6	7	6
1.4–1.6	171	83	77	146	52	4	6	3	3
1.6–1.8	-	55	41	38	128	123	101	18	9
1.8–2.0	-	4	29	31	43	99	121	77	66
2.0–2.2	-	-	29	7	5	2	-	57	68
>2.2	-	-	9	-	-	-	-	74	84
At 1.0 cm depth									
<1.2	21	12	8	4	4	3	3	1	1
1.2–1.4	46	47	32	7	5	6	6	7	6
1.4–1.6	170	113	58	130	23	4	6	3	3
1.6–1.8	-	61	67	54	154	95	58	12	9
1.8–2.0	-	4	31	35	46	127	164	38	28
2.0–2.2	-	-	32	7	5	2	-	84	80
>2.2	-	-	9	-	-	-	-	92	110
At 2.0 cm depth									
<1.2	21	12	8	4	4	3	3	1	1
1.2–1.4	50	35	27	7	5	6	6	7	7
1.4–1.6	166	105	38	112	13	4	6	5	2
1.6–1.8	-	81	62	70	159	67	46	11	9
1.8–2.0	-	4	57	35	51	155	176	27	30
2.0–2.2	-	-	36	9	5	2	-	55	46
>2.2	-	-	9	-	-	-	-	131	142

**Table 4:** Pairwise comparison of the efficacy of NPs with different compositions in terms of DEF.

	Cu	ZrO_2_	Ag	CeO_2_	Gd_2_O_3_	Tm_2_O_3_	HfO_2_	Bi_2_O_3_	Au
Cu		55–87%	72–85%	61–67%	100%	100%	100%	100%	100%
ZrO_2_	13–45%		85–91%	63–82%	96–99%	100%	100%	100%	100%
Ag	15–28%	9–15%		23–47%	46–70%	67–76%	68–74%	99%	99%
CeO_2_	33–39%	18–37%	53–73%		95%	93–94%	74–78%	98%	99%
Gd2O_3_	0%	1–4%	30–54%	5%		73–87%	72–85%	94%	98%
Tm_2_O_3_	0%	0%	24–33%	6–7%	13–27%		64–78%	95–96%	98%
HfO_2_	0%	0%	26–32%	12–16%	15–28%	22–36%		97–98%	100%
Bi_2_O_3_	0%	0%	1%	2%	6%	4–5%	2–3%		81–87%
Au	0%	0%	1%	1%	2%	2%	0%	13–19%	

Materials located horizontally must be compared with those located vertically. The table shows the percentage of the total number of the studied spectra, where the material from the top row of the table dominates. Two numbers in a cell indicate a change in the effect with depth.

**Table 5:** Characteristics of substances forming the studied high-*Z* nanoparticles (NPs).

Number	Formula	Mass Composition	Atomic Number (*Z*)	Effective Atomic Number (*Z*)
1	Cu	Cu: 100%	*Z*_Cu_ = 29	29
2	ZrO_2_	Zr: 74% O: 26%	*Z*_Zr_ = 40 *Z*_O_ = 8	36.34
3	Ag	Ag: 100%	*Z*_Ag_ = 47	47
4	CeO_2_	Ce: 81.4% O: 18.6%	*Z*_Ce_ = 58 *Z*_O_ = 8	59.64
5	Gd_2_O_3_	Gd: 86.8% O: 13.2%	*Z*_Gd_ = 64 *Z*_O_ = 8	62.28
6	Tm_2_O_3_	Tm: 87.6% O: 12.4%	*Z*_Tm_ = 69 *Z*_O_ = 8	67.28
7	HfO_2_	Hf: 84.8% O: 15.2%	*Z*_Hf_ = 72 *Z*_O_ = 8	67.99
8	Ta_2_O_5_	Ta: 81.9% O: 18.1%	*Z*_Ta_ = 73 *Z*_O_ = 8	68.12
9	Bi_2_O_3_	Bi: 89.7% O: 10.3%	*Z*_Bi_ = 83 *Z*_O_ = 8	81.26
10	Pt	Pt: 100%	*Z*_Pt_ = 78	78
11	Au	Au: 100%	*Z*_Au_ = 79	79

## Data Availability

No new data were created or analyzed in this study. Data sharing is not applicable to this article.

## Supplementary Materials

The following are available online at https://www.mdpi.com/article/10.3390/ijms22116030/s1, Table S1–S29: Characteristics of X-ray tubes and spectra generated at a fixed 30–300 kVp voltages.
