# Supplementary material for: Impact of the Spectral Composition of Kilovoltage X-rays on High-Z Nanoparticle-Assisted Dose Enhancement
Source: Int J Mol Sci. 2021 Jun 2;22(11):6030. doi: 10.3390/ijms22116030 (PMC8199749; doi:10.3390/ijms22116030)
Supplement: Supplementary file 1 [file ijms-22-06030-s001.zip › ijms-1215065-supplementary.pdf]

Supporting information for the manuscript

Impact of the spectral composition of kilovoltage X-rays on high-*Z* nanoparticle-assisted dose enhancement

Maria A. Kolyvanova [1,2], Alexandr V. Belousov [1], Grigorii A. Krusanov [1], Alexandra K. Isagulieva [1,3], Kirill V. Morozov[4], Maria E. Kartseva[5], Magomet H. Salpagarov[2], Pavel V. Krivoshapkin[7], Olga V. Dement`eva[5], Victor M. Rudoy[5], Vladimir N. Morozov [2,*]

1 – State Research Center-Burnasyan Federal Medical Biophysical Center of Federal Medical Biological Agency, 123098 Moscow, Russia; kolyvanova@physics.msu.ru (M.A.K.); belousovav@physics.msu.ru (A.V.B.); krusanov@physics.msu.ru (G.A.K.); kia2303@yandex.ru (A.K.I.); morozov.kv15@physics.msu.ru (K.V.M.); magometonco@mail.ru (M.H.S.)

2 – Emanuel Institute of Biochemical Physics, Russian Academy of Sciences, 119334 Moscow, Russia;

3 – Gause Institute of New Antibiotics, 119021 Moscow, Russia

4 – Department of Physics, Lomonosov Moscow State University, 119234 Moscow, Russia

5 – Frumkin Institute of Physical Chemistry and Electrochemistry, Russian Academy of Sciences, 119071 Mos-cow, Russia; Russia; maryakar@mail.ru (M.E.K.); dema_ol@mail.ru (O.V.D. and V.M.R.)

6 – ITMO University, St. Petersburg 191002, Russia; krivoshapkin@scamt-itmo.ru (P.V.K.)

** Correspondence: morozov.v.n@mail.ru; Tel.: +7-985-117-36-03*

Table S1. Characteristics of X-ray tubes and spectra generated at a fixed 30 kVp voltage.

Spectrum number	Anode material	Filtration system	Effective energy, keV	Average energy, keV
30_1 (60)	W	3.0 mm Be + 0.5 mm Al	16.11	19.47
30_2 (64)	W	0.49 mm Al	15.81	19.27
30_3 (85)	W	0.8 mm C + 0.05 mm Pd	18.00	19.85
30_4 (86)	Mo	0.8 mm C + 0.05 mm Mo	15.73	17.15
30_5 (89)	W	0.5 mm Be + 1.2 mm Al	19.26	21.66
30_6 (90)	Mo	0.5 mm Be + 0.03 mm Mo	14.22	16.51
30_7 (91)	Rh	0.5 mm Be + 0.025 mm Rh	15.25	18.00
30_8 (92)	Mo	0.5 mm Be + 0.025 mm Rh	15.23	17.72
30_9 (115)	Mo	0.03 mm Mo + 4.0 mm Be	21.86	24.84
30_10 (179)	W	0.52 mm Al	16.04	19.39
30_11 (183)	W	0.208 mm Al	12.44	16.98
30_12 (184)	W	0.5 mm Al	15.76	19.27
30_13 (185)	Mo	0.05 mm Ag	17.71	19.98
30_14 (186)	Mo	0.05 mm Mo	15.42	17.10
30_15 (187)	W	0.06 mm Mo	15.90	17.28
30_16 (188)	Mo	0.03 mm Mo	14.10	16.36
30_17 (190)	Mo	0.025 mm Rh	15.08	17.61
30_18 (191)	Mo	1.0 mm Al	18.03	20.46
30_19 (192)	Rh	0.025 mm Rh	15.05	17.84
30_20 (193)	Rh	1.0 mm Al	18.27	20.73

Table S2. Characteristics of X-ray tubes and spectra generated at a fixed 35 kVp voltage.

Spectrum number	Anode material	Filtration system	Effective energy, keV	Average energy, keV
35_1 (52)	W	0.47 mm Al	16.04	20.48
35_2 (250)	Rh	1.0 mm Al	19.26	22.45

35_3 (252)	Rh	0.025 mm Rh	15.56	18.81
35_4 (266)	Mo	0.03 mm Mo	15.99	17.73
35_5 (267)	Mo	0.025 mm Rh	16.51	18.22
35_6 (269)	Mo	0.5 mm Al	16.74	19.21
35_7 (270)	Mo	1.0 mm Al	16.70	19.21
35_8 (271)	W	0.05 mm Ag	19.11	21.57
35_9 (272)	W	0.5 mm Al	17.23	22.20
35_10 (273)	W	2.7 mm Al	23.90	26.31
35_11 (274)	W	0.05 mm Rh	18.60	20.64
35_12 (275)	W	0.7 mm Al	21.27	23.94
35_13 (276)	Mo	3.0 mm Al	21.08	24.61
35_14 (278)	Rh	3.0 mm Al	21.80	24.30

Table S3. Characteristics of X-ray tubes and spectra generated at a fixed 40 kVp voltage.

Spectrum number	Anode material	Filtration system	Effective energy, keV	Average energy, keV
40_1 (18)	W	0.04 mm Pd	18.87	22.27
40_2 (53)	W	0.56 mm Al	17.63	23.23
40_3 (59)	W	3.0 mm Be + 0.8 mm Al	19.51	24.46
40_4 (65)	W	0.49 mm Al	16.93	22.66
40_5 (128)	Mo	0.025 mm Rh + 2.0 mm Al	23.11	27.22
40_6 (135)	W	2.5 mm Al	25.33	28.46
40_7 (136)	W	0.5 mm Al	18.18	24.13
40_8 (138)	W	0.05 mm Rh	19.07	21.98
40_9 (141)	Rh	3.0 mm Al	24.29	27.62
40_10 (142)	Mo	3.0 mm Al	22.69	27.23
40_11 (143)	W	0.05 mm Ag	19.72	22.74
40_12 (145)	W	0.05 mm Ag + 2.0 mm Al	22.89	26.04
40_13 (242)	Mo	1.0 mm Al	19.43	23.79
40_14 (243)	Rh	1.0 mm Al	19.95	24.04
40_15 (244)	Rh	0.025 mm Rh	19.42	23.79
40_16 (245)	Mo	0.03 mm Mo	19.44	23.79
40_17 (246)	Mo	0.025 mm Mo	14.22	18.16
40_18 (247)	Rh	0.025 mm Rh + 2.0 mm Al	21.96	25.36
40_19 (248)	W	0.8 mm Al	19.31	24.25
40_20 (249)	W	4.0 mm Be + 0.5 mm Al	17.20	22.87
40_21 (251)	W	1.0 mm Al	20.33	24.99
40_22 (279)	W	0.06 mm Mo	17.14	21.18

Table S4. Characteristics of X-ray tube and spectrum generated at a fixed 45 kVp voltage.

Spectrum number	Anode material	Filtration system	Effective energy, keV	Average energy, keV
45_1 (54)	W	0.74 mm Al	19.50	25.84

Table S5. Characteristics of X-ray tubes and spectra generated at a fixed 50 kVp voltage.

Spectrum number	Anode material	Filtration system	Effective energy, keV	Average energy, keV
50_1 (31)	W	0.5 mm Be	6.22	15.53
50_2 (55)	W	1.01 mm Al	21.64	28.31
50_3 (58)	W	3.0 mm Be + 1.0 mm Al	21.85	28.56
50_4 (66)	W	0.96 mm Al	6.68	21.59
50_5 (84)	W	0.8 mm C + 1.0 mm Ba	19.59	24.79
50_6 (88)	Sb	0.8 mm C + 0.2 mm Sb	24.97	27.53
50_7 (93)	W	3.0 mm Be + 1.65 mm Al	24.58	30.46
50_8 (130)	W	0.05 mm Rh	19.77	25.66
50_9 (131)	Mo	3.0 mm Al	24.60	31.17
50_10 (132)	Rh	3.0 mm Al	24.34	30.07

50_11 (206)	W	1.0 mm Al	21.54	28.37
50_12 (221)	W	0.25 mm Al	14.45	23.37
50_13 (222)	W	0.04 mm Ag	18.74	24.07
50_14 (223)	W	0.5 mm Al	18.00	25.82
50_15 (224)	W	2.5 mm Al	26.91	32.06
50_16 (225)	W	0.04 mm Ag + 2.0 mm Al	24.51	30.40
50_17 (226)	W	0.04 mm Pd	18.54	23.91
50_18 (227)	W	0.04 mm Pd + 2.0 mm Al	24.06	30.91
50_19 (228)	W	0.05 mm Mo	16.64	23.17
50_20 (229)	W	0.05 mm Mo + 2.0 mm Al	25.24	34.15
50_21 (230)	W	0.06 mm Mo	17.09	23.53
50_22 (231)	W	0.06 mm Mo + 2.0 mm Al	25.57	34.58
50_23 (232)	W	1.65 mm Al	24.45	30.35
50_24 (233)	W	2.46 mm Al	26.81	32.00
50_25 (234)	Mo	0.03 mm Mo	15.25	20.68
50_26 (235)	Mo	0.03 mm Mo + 2.0 mm Al	23.51	31.75
50_27 (236)	Mo	0.025 mm Rh	16.33	21.54
50_28 (237)	Mo	0.025 mm Rh + 2.0 mm Al	23.25	30.22
50_29 (238)	Rh	0.025 mm Rh	16.58	21.95
50_30 (239)	Rh	0.025 mm Rh + 2.0 mm Al	23.37	29.84
50_31 (240)	Rh	1.0 mm Al	20.69	26.79
50_32 (241)	Mo	1.0 mm Al	20.18	26.68
50_33 (280)	W	0.05 mm Ag	20.44	25.93
50_34 (281)	W	0.05 mm Ag + 2.0 mm Al	24.84	31.21
50_35 (282)	W	0.05 mm Rh + 2.0 mm Al	24.73	32.72
50_36 (283)	W	4.0 mm Al	29.76	34.16

Table S6. Characteristics of X-ray tube and spectrum generated at a fixed 55 kVp voltage.

Spectrum number	Anode material	Filtration system	Effective energy, keV	Average energy, keV
55_1 (29)	W	1.0 mm Be + 0.78 mm Al	6.30	16.61

Table S7. Characteristics of X-ray tubes and spectra generated at a fixed 60 kVp voltage.

Spectrum number	Anode material	Filtration system	Effective energy, keV	Average energy, keV
60_1 (27)	W	0.5 mm Be	6.38	17.68
60_2 (120)	W	3.2 mm Al	30.48	36.99
60_3 (121)	W	3.9 mm Al	31.37	37.58
60_4 (123)	W	0.3 mm Cu + 4.0 mm Al	32.68	44.18
60_5 (125)	W	18.7 mm Al	40.57	45.39
60_6 (127)	W	0.6 mm Cu + 4.0 mm Al	44.22	47.35
60_7 (152)	W	0.4 mm Be	6.15	18.31
60_8 (196)	W	2.68 mm Al	28.76	35.82
60_9 (199)	W	0.4 mm Be + 0.015 mm V	6.15	18.32
60_10 (200)	W	0.4 mm Be + 0.0015 mm Fe	6.37	18.41
60_11 (201)	W	0.4 mm Be + 0.005 mm Mn	6.48	20.30
60_12 (202)	W	0.4 mm Be + 0.015 mm Ni	15.89	28.36
60_13 (203)	W	0.4 mm Be + 0.075 mm Zr	15.89	28.36
60_14 (277)	W	4.0 mm Al + 0.6 mm Cu	44.81	47.43

Table S8. Characteristics of X-ray tubes and spectra generated at a fixed 70 kVp voltage.

Spectrum number	Anode material	Filtration system	Effective energy, keV	Average energy, keV
70_1 (44)	W	1.65 mm Al	23.61	34.25
70_2 (57)	W	3.0 mm Be + 4.0 mm Al	33.44	41.29
70_3 (75)	W	4.0 mm Al	33.51	41.15
70_4 (118)	W	2. 83 mm Al + 1.5 mm Cu	47.94	57.18

70_5 (220)	W	2.83 mm Al	30.78	39.28

Table S9. Characteristics of X-ray tubes and spectra generated at a fixed 75 kVp voltage.

Spectrum number	Anode material	Filtration system	Effective energy, keV	Average energy, keV
75_1 (94)	W	3.0 mm Be + 2.4 mm Al	27.44	38.26
75_2 (219)	W	2.4 mm Al	30.06	39.98

Table S10. Characteristics of X-ray tubes and spectra generated at a fixed 80 kVp voltage.

Spectrum number	Anode material	Filtration system	Effective energy, keV	Average energy, keV
80_1 (25)	W	w/o filtration	2.48	19.85
80_2 (35)	W	0.8 mm Al	48.21	53.39
80_3 (67)	W	1.02 mm Al	24.46	37.07
80_4 (96)	W	3.0 mm Al	32.13	47.58
80_5 (116)	W	7.2 mm Al	40.20	47.93
80_6 (204)	W	2.0 mm Cu + 4.0 mm Al	54.39	64.98
80_7 (205)	W	0.5 mm Cu + 4.0 mm Al	44.88	56.40
80_8 (207)	W	2.99 mm Al	32.48	42.77

Table S11. Characteristics of X-ray tubes and spectra generated at a fixed 90 kVp voltage.

Spectrum number	Anode material	Filtration system	Effective energy, keV	Average energy, keV
90_1 (22)	W	3.0 mm Al	7.25	24.68
90_2 (23)	W	2.5 mm Al	7.25	24.67
90_3 (24)	W	2.0 mm Al	7.25	24.67
90_4 (214)	W	6.36 mm Al	34.46	46.15

Table S12. Characteristics of X-ray tubes and spectra generated at a fixed 100 kVp voltage.

Spectrum number	Anode material	Filtration system	Effective energy, keV	Average energy, keV
100_1 (19)	W	1.0 mm Be + 2.0 mm Al	7.33	26.93
100_2 (20)	W	1.0 mm Be + 0.78 mm Cu	7.33	26.92
100_3 (21)	W	2.2 mm Be + 1.0 mm Cu	7.33	26.92
100_4 (37)	W	1.0 mm Cu + 1.8 mm Al	27.82	47.80
100_5 (43)	W	2.1 mm Al	31.95	46.27
100_6 (56)	W	3.0 mm Be + 4.5 mm Al	39.65	51.30
100_7 (68)	W	2.03 mm Al	31.68	46.07
100_8 (76)	W	4.5 mm Al	39.63	51.09
100_9 (95)	W	3.0 mm Be + 3.1 mm Al	30.25	45.17
100_10 (103)	W	0.5 mm Cu + 1.0 mm Al	14.32	34.45
100_11 (104)	W	0.6 mm Cu + 4.0 mm Al	53.26	65.00
100_12 (194)	W	4.4 mm Al	39.10	50.89
100_13 (195)	W	3.0 mm Be + 3.4 mm Al	38.81	50.75
100_14 (197)	W	3.506 mm Al	36.76	49.42
100_15 (212)	W	0.2 mm Cu + 3.4 mm Al	42.11	57.55
100_16 (216)	W	3.1 mm Al	35.57	48.63
100_17 (217)	W	3.36 mm Al	36.24	49.14
100_18 (218)	W	0.15 mm Cu + 3.9 mm Al	39.98	56.37
100_19 (255)	W	w/o filtration	2.75	23.90
100_20 (256)	W	0.5 mm Al	20.97	38.80
100_21 (257)	W	1.0 mm Al	25.81	42.04
100_22 (265)	W	2.0 mm Cu + 3.36 mm Al	63.33	73.17

Table S13. Characteristics of X-ray tubes and spectra generated at a fixed 110 kVp voltage.

Spectrum number	Anode material	Filtration system	Effective energy, keV	Average energy, keV
110_1 (113)	W	1.3 mm Cu + 5.5 mm Al	65.88	75.62
110_2 (114)	W	1.5 mm Cu + 2.0 mm Al	66.90	76.27
110_3 (211)	W	2.0 mm Cu + 4.0 mm Al	71.40	79.41

Table S14. Characteristics of X-ray tubes and spectra generated at a fixed 120 kVp voltage.

Spectrum number	Anode material	Filtration system	Effective energy, keV	Average energy, keV
120_1 (17)	W	2.5 mm Al	7.46	31.23
120_2 (38)	W	0.3 mm Cu + 1.1 mm Al	30.22	52.53
120_3 (39)	W	1.0 mm Cu + 0.2 mm Al	63.91	75.49
120_4 (69)	W	4.0 mm Al	40.82	55.04
120_5 (77)	W	6.0 mm Al	28.03	47.13
120_6 (208)	W	0.29 mm Cu + 3.7 mm Al	49.91	65.50
120_7 (209)	W	3.73 mm Al	40.22	54.58
120_8 (263)	W	1.0 mm Sn + 5.0 mm Cu + 4.0 mm Al	67.19	71.96

Table S15. Characteristics of X-ray tubes and spectra generated at a fixed 125 kVp voltage.

Spectrum number	Anode material	Filtration system	Effective energy, keV	Average energy, keV
125_1 (210)	W	3.3 mm Cu + 3.6 mm Al	84.55	91.71
125_2 (213)	W	4.2 mm Cu	88.48	94.15
125_3 (215)	W	0.1 mm Cu + 2.5 mm Al	46.87	59.51

Table S16. Characteristics of X-ray tube and spectrum generated at a fixed 135 kVp voltage.

Spectrum number	Anode material	Filtration system	Effective energy, keV	Average energy, keV
135_1 (182)	W	0.222 mm Cu + 2.302 mm Al	48.79	66.51

Table S17. Characteristics of X-ray tube and spectrum generated at a fixed 140 kVp voltage.

Spectrum number	Anode material	Filtration system	Effective energy, keV	Average energy, keV
140_1 (78)	W	9.0 mm Al	53.80	65.21

Table S18. Characteristics of X-ray tubes and spectra generated at a fixed 150 kVp voltage.

Spectrum number	Anode material	Filtration system	Effective energy, keV	Average energy, keV
150_1 (14)	W	0.6 mm Cu	7.64	37.26
150_2 (15)	W	0.5 mm Ti + 2.0 mm H_2O	7.65	37.26
150_3 (16)	W	2.2 mm Be + 4.0 mm Al	7.65	37.26
150_4 (70)	W	0.3 mm Cu + 1.2 mm Al	61.17	71.46
150_5 (79)	W	0.5 mm Cu + 4.0 mm Al	63.66	77.73
150_6 (97)	W	1.5 mm Al	33.65	54.82
150_7 (98)	W	0.1 mm Cu + 0.3 mm Al	35.87	61.43
150_8 (105)	W	0.2 mm Cu + 0.5 mm Al	46.90	67.47
150_9 (106)	W	0.2 mm Cu + 5.0 mm Al	53.23	70.97
150_10 (111)	W	2.2 mm Cu	87.96	95.77
150_11 (112)	W	1.1 mm Cu	73.86	85.65
150_12 (170)	W	0.3 mm Cu + 4.4 mm Al	56.94	73.43
150_13 (171)	W	4.38 mm Al	46.03	61.91

150_14 (172)	W	2.5 mm Sn + 4.0 mm Al	103.39	115.69
150_15 (173)	W	1.0 mm Sn + 4.38 mm Al	87.81	106.42
150_16 (177)	W	0.53 mm Cu + 4.0 mm Al	22.38	52.93
150_17 (178)	W	1.01 mm Cu	21.13	47.89
150_18 (180)	W	0.9 mm Cu + 1.0 mm Al	71.23	83.50
150_19 (181)	W	0.5 mm Cu + 4.5 mm Al	63.37	77.86
150_20 (253)	W	49.4 mm Al	76.05	81.44

Table S19. Characteristics of X-ray tube and spectrum generated at a fixed 160 kVp voltage.

Spectrum number	Anode material	Filtration system	Effective energy, keV	Average energy, keV
160_1 (13)	W	1.0 mm Be	7.71	39.18

Table S20. Characteristics of X-ray tubes and spectra generated at a fixed 180 kVp voltage.

Spectrum number	Anode material	Filtration system	Effective energy, keV	Average energy, keV
180_1 (12)	W	0.15 mm Cu + 2.5 mm Al	7.79	42.88
180_2 (42)	W	0.35 mm Cu + 1.5 mm Al	69.24	79.56
180_3 (99)	W	5.5 mm Be + 1.0 mm Al	33.39	58.50
180_4 (100)	W	3.0 mm Be + 2.3 mm Al	42.12	63.38
180_5 (169)	W	0.53 mm Cu + 6.0 mm Al	71.15	86.14

Table S21. Characteristics of X-ray tubes and spectra generated at a fixed 200 kVp voltage.

Spectrum number	Anode material	Filtration system	Effective energy, keV	Average energy, keV
200_1 (8)	W	0.8 mm Be	7.85	46.40
200_2 (9)	W	7.0 mm Be + 0.5 mm Cu	7.84	46.35
200_3 (10)	W	7.0 mm Be + 0.6 mm Cu	7.86	46.38
200_4 (11)	W	0.6 mm Cu	7.85	46.40
200_5 (71)	W	1.2 mm Cu + 4.0 mm Al	97.07	101.23
200_6 (80)	W	1.0 mm Cu + 4.0 mm Al	86.76	98.73
200_7 (149)	W	1.15 mm Cu +4.0 mm Al	20.42	67.93
200_8 (155)	W	2.0 mm Sn + 4.0 mm Al	27.16	66.50
200_9 (156)	W	0.99 mm Cu + 6.0 mm Al	31.96	72.73
200_10 (161)	W	0.5 mm Cu + 2.0 mm Al	24.47	60.85
200_11 (262)	W	1.0 mm Pb + 3.0 mm Sn + 2.0 mm Cu + 4.0 mm Al	131.10	140.03
200_12 (268)	W	4.0 mm Al + 2.0 mm Cu + 3.0 mm Sn + 1.0 mm Pb	159.66	161.53
200_13 (P)	W	1.0 mm Al + 1.0 mm Cu	88.09	94.17
200_14 (R)	W	2.5 mm Glass + 1.5 mm Al	53.67	64.72
200_15 (X)	W	1.0 mm Al + 0.45 mm Cu	72.62	82.69

Table S22. Characteristics of X-ray tubes and spectra generated at a fixed 205 kVp voltage.

Spectrum number	Anode material	Filtration system	Effective energy, keV	Average energy, keV
205_1 (101)	W	1.0 mm Be + 0.1 mm Al	44.86	67.68
205_2 (102)	W	5.5 mm Be + 0.1 mm Al	18.11	54.75

Table S22. Characteristics of X-ray tubes and spectra generated at a fixed 210 kVp voltage.

Spectrum number	Anode material	Filtration system	Effective energy, keV	Average energy, keV
210_1 (107)	W	1.0 mm Be + 3.4 mm Al	62.83	83.02

210_2 (108)	W	4.8 mm Be + 4.0 mm Al	55.41	79.04

Table S23. Characteristics of X-ray tubes and spectra generated at a fixed 220 kVp voltage.

Spectrum number	Anode material	Filtration system	Effective energy, keV	Average energy, keV
220_1 (144)	W	7.1 mm Be + 0.3 mm Cu	23.67	68.22
220_2 (146)	W	4.1 mm Be + 0.2 mm Cu	56.20	80.26
220_3 (166)	W	0.5 mm Cu	72.63	91.19
220_4 (167)	W	4.05 mm Al	53.31	73.58

Table S24. Characteristics of X-ray tubes and spectra generated at a fixed 225 kVp voltage.

Spectrum number	Anode material	Filtration system	Effective energy, keV	Average energy, keV
225_1 (7)	W	0.1 mm Be	7.93	50.62
225_2 (41)	W	1.0 mm Al + 0.9 mm Cu	3.82	50.25
225_3 (139)	W	0.5 mm Cu + 4.0 mm Al	18.01	72.73

Table S25. Characteristics of X-ray tube and spectrum generated at a fixed 240 kVp voltage.

Spectrum number	Anode material	Filtration system	Effective energy, keV	Average energy, keV
240_1 (8)	W	4.1 mm Be + 0.6 mm Cu	16.95	74.41

Table S26. Characteristics of X-ray tubes and spectra generated at a fixed 250 kVp voltage.

Spectrum number	Anode material	Filtration system	Effective energy, keV	Average energy, keV
250_1 (6)	W	0.4 mm Sn + 0.35 mm Cu	7.97	54.63
250_2 (72)	W	2.91 mm Cu + 1.02 mm Al	133.51	131.41
250_3 (81)	W	1.6 mm Cu + 4.0 mm Al	113.76	120.39
250_4 (133)	W	0.01 mm Cu + 0.02 mm Al	22.14	70.01
250_5 (134)	W	0.01 mm Al + 0.01 mm Sn	24.55	72.86
250_6 (261)	W	3.0 mm Pb + 2.0 mm Sn + 4.0 mm Al	179.65	188.12

Table S27. Characteristics of X-ray tubes and spectra generated at a fixed 260 kVp voltage.

Spectrum number	Anode material	Filtration system	Effective energy, keV	Average energy, keV
260_1 (109)	W	5.5 mm Be + 0.2 mm Al	25.92	65.22
260_2 (110)	W	6.5 mm Be + 0.3 mm Al	29.78	66.41

Table S28. Characteristics of X-ray tube and spectrum generated at a fixed 280 kVp voltage.

Spectrum number	Anode material	Filtration system	Effective energy, keV	Average energy, keV
280_1 (82)	W	4.0 mm Al + 3.0 mm Cu	141.76	141.93

Table S29. Characteristics of X-ray tubes and spectra generated at a fixed 300 kVp voltage.

Spectrum number	Anode material	Filtration system	Effective energy, keV	Average energy, keV
300_1 (5)	W	0.3 mm Be + 0.5 mm Cu	7.98	62.24
300_2 (73)	W	2.9 mm Cu + 1.02 mm Al	149.34	144.93
300_3 (74)	W	4.0 mm Sn + 4.0 mm Al	191.80	186.84
300_4 (119)	W	2.5 mm Cu + 4.0 mm Al	10.25	66.94
300_5 (122)	W	6.5 mm Sn + 4.0 mm Al	30.59	75.09

300_6 (124)	W	4.1 mm Be + 0.4 mm Cu	83.06	102.77
300_7 (126)	W	1.1 mm Be + 0.1 mm Cu	29.56	72.25
300_8 (129)	W	4.1 mm Al	63.21	85.79
300_9 (254)	W	3.0 mm Cu + 4.0 mm Al	148.12	147.06
300_10 (260)	W	5.0 mm Pb + 3.0 mm Sn + 4.0 mm Al	234.76	235.62
